# System dynamics modelling to engage community stakeholders in addressing water and sugar sweetened beverage consumption

**DOI:** 10.1186/s12966-022-01363-4

**Published:** 2022-09-10

**Authors:** Andrew D. Brown, Kristy A. Bolton, Brydie Clarke, Penny Fraser, Janette Lowe, Jake Kays, Peter S. Hovmand, Steven Allender

**Affiliations:** 1 Global Centre for Preventive Health and Nutrition, Institute for Health Transformation, Deakin University, Geelong, Victoria Australia; 2 Institute for Physical Activity and Nutrition, Deakin University, Geelong, Victoria Australia; 3 Southern Grampians Glenelg Primary Care Partnership, Hamilton, Victoria Australia; 4 Center for Community Health Integration, School of Medicine and Case School of Engineering, Case Western Reserve University, Cleveland, OH USA

**Keywords:** System dynamics, Group model building, Sugar sweetened beverages, Water consumption, Community-based participatory research

## Abstract

**Background:**

Participatory approaches to develop community-based system dynamic**s** models to tackle complexity are promising**,** but research is needed in how simulation models can be developed with community stakeholders to yield significant system insights. This study presents the results of a community-based system dynamics modelling process to increase water consumption and decrease sugar sweetened beverage consumption in Portland, Victoria, a regional town in Australia.

**Methods:**

A series of group model building workshops with 11 community stakeholders addressing the topic of water and sugar sweetened beverage consumption was conducted in Portland. A simulating system dynamics model was built with stakeholders to inform action planning.

**Results:**

A system dynamics model was created to provide insight into water and sugar sweetened beverage consumption in Portland. The model included six feedback loops describing the causal effects of sugar sweetened beverage consumption habits and norms, water taste, water consumption norms, public water availability, and public health benefits. For example, the sugar sweetened beverage consumption norm loop modelled how people overestimating others’ consumption may motivate an increase in their own consumption, feeding back and further amplifying an increase in sugar sweetened beverage consumption. The model contributed to the foundation of a strong partnership to improve the taste of water and educate the public on water consumption.

**Conclusions:**

Engaging stakeholders in system dynamics modelling about water and sugar sweetened beverage consumption increased engagement and collaboration to address the problem among community stakeholders.

**Supplementary Information:**

The online version contains supplementary material available at 10.1186/s12966-022-01363-4.

## Background

Sugar sweetened beverage (SSB) consumption is associated with the development of overweight and obesity [1], cardiovascular disease [2], metabolic syndrome [3] and dental decay [4]. In 2014 the World Health Organization called for a population level shift from SSBs to water as a strategy to achieve the daily sugar consumption target of six or less teaspoons per day [5]. Current efforts to shift from SSBs to water may include policies (e.g. SSB tax) [6, 7], behaviour change techniques [8] or environmental strategies [9]. Reasons for SSB consumption are complex and driven by multiple interacting factors [10]. Consequently singular actions have provided only modest population level impacts indicating that multiple, coordinated initiatives are needed [11, 12]. There are some studies that have evaluated combined individual level and environment or policy level interventions, but more such studies are needed to build evidence about the effectiveness of combining interventions [12].

Combining interventions to shift SSB consumption to water consumption at multiple levels is complex due to the interactions of those interventions. For example, because of the interactions of different settings (such as schools, workplaces, and homes), efforts to reduce SSB consumption in one setting could be undermined in another [13]. Interventions also need to be tailored to and supported by the local community [14]. Additionally, environmental and policy interventions can come with unintended consequences, where changes in individual behaviour or actions of corporations to influence behaviour may undermine intervention outcomes. These various interactions create a need to consider combining mutually reinforcing behavioural and policy or environmental interventions [12].

Public health engages methods from system science to better understand the complexity of health issues arising from multiple interacting components and unintended consequences to develop comprehensive interventions [15–18]. System dynamics (SD), a subset of systems science, articulates how parts of a system interact over time through feedback and how system structure (the parts of a system and their relationships that work together to achieve a shared purpose [19]) results in observed outcomes [15, 20, 21]. The concept of feedback loops in system dynamics offers the possibility to consider the complex interactions between individual, policy, and environmental actions to shift SSB consumption to water consumption.

Feedback loops are causal explanations of change in a system where change is caused by two or more variables that mutually affect one another. In reinforcing loops, change is amplified over time in all variables involved whereas in balancing loops, the pattern of interaction between variables counteracts change and favours stability [22]. SD uses stock and flow diagrams to visually depict the interacting reinforcing and balancing loops that may impact system behaviour(s) of interest. People have a limited ability to infer the behaviour of a set of feedback loops shown in a diagram, which can make it difficult to translate a visual depiction of a system into effective action [23]. Formal computer simulation (SD simulation models) quantifies relationships hypothesised in stock and flow diagrams and provides insight into possible system behaviour given hypothesised system structure [24]. These models can be informed by published research, literature, available data, and local knowledge of people in the system.

Previous SD models have been critiqued for being expert led (by academics or actors external to a particular setting) resulting in a less context specific understanding and lack of stakeholder buy-in, hindering decision making and implementation [25, 26]. Examples of participatory approaches to apply systems thinking tools in community settings appear to provide rich insights into complex issues and potentially strengthen interventions [15, 27, 28]. The Whole of Systems Trial of Prevention Strategies for Childhood Obesity (WHO STOPS) [18] intervention intended to test the impact of developing community capacity to apply systems thinking in the prevention of childhood obesity in Southwestern Victoria, Australia.

Shifting SSB consumption to water consumption was one key area identified by Portland, a WHO STOPS pilot community because of the links between obesity and sugar consumption [29]. Despite the emergence of strategies globally to target sugar reduction and increase water consumption, [11, 12, 30] SSB consumption levels remain high [31] and more than half of Australians consume more than the daily recommended amount [32]. The latest Australian data show that 42% of Australians aged 2 years and over, or 9 million people, consume sugar-sweetened beverages, with males more likely to consume these products than females (39% compared with 29%) [32]. People in rural areas are more likely to consume SSBs regularly than in metropolitan areas [33].

This study engaged residents of Portland to test a community-based application of SD modelling to engage with the complexity of shifting SSB consumption to water consumption. This paper describes the development of a community-based SD simulation model of water and SSB consumption in a regional community in Victoria, Australia. We demonstrate how the process engaged stakeholders and prompted them to consider the complexity of SSB and water consumption and to commit to taking action.

## Methods

### Aim

Community stakeholders in a regional town, Portland, Victoria, Australia working on childhood obesity prevention, had previously indicated improving water taste as a priority action to increase water consumption and decrease SSB consumption [29]. However, given the major infrastructure costs involved in improving water taste, stakeholders and researchers aimed to explore further the interacting drivers of water consumption and SSB consumption to decide on the right combination of actions.

### Study design

This study used community-based system dynamics [25] to build a SD model of the local drivers that influence water and SSB consumption and begin to form a shared action plan.

### Setting

The model was developed with key stakeholders over three workshops between May 2016 and February 2017. Participants were the same 11 key stakeholders from Portland with an interest or role in consumption of SSBs or water and included representatives from the Primary Care Partnership, local government, health service, sporting clubs, the local water authority, and community members, with diverse perspectives on the taste of the water in Portland. The workshops were held as part of an ongoing effort led by a local health organisation, who identified and invited key stakeholders who would have both insight into the system and power to make change.

### Data collection

Three group model building (GMB) [25, 34] workshops were conducted (in May 2016, August 2016, and February 2017) comprising “scripted” activities to work with key stakeholders to first develop an informal map (a stock and flow diagram) and then iterate on formal computer simulation models [22]. GMB is particularly useful for SD model development, as this process uses systems language and concepts when capturing stakeholder knowledge and experiences to better understand complex problems [29]. The scripted activities consist of a set of facilitated structured group exercises [35, 36], which were used to establish the boundaries for the model, identify key variables and the connections between the variables, and review and refine maps. The focal issue for the GMB workshops was declining water consumption and increasing SSB consumption within the residents of Portland. SD simulation models were developed using Vensim [37].

#### First workshop

At the first 90-minute workshop, participants brainstormed the different variables that affect water and SSB consumption locally using the “Graphs over Time” GMB script [36, 38]. In this activity, participants also consider how variables change over time and draw a graph to represent the variable’s past behaviour and feared and hoped future behaviour. Participants shared their individually brainstormed ideas and then prioritised the most important variables as a group. The group then worked together to draw causal arrows between factors to collectively develop an initial stock and flow diagram visually depicting the hypothesised system of interest using the “Initiating and Elaborating a Causal Loop Diagram or Stock and Flow Model” script [36].

#### Second workshop

After the first workshop, the model was parametrised using the descriptions given by stakeholders in the first workshop and from quantitative measures of cause-and-effect relationships in the published research literature [39, 40]. The participants from workshop one then reconvened for a second 90-minute workshop.

An overview of the model was presented and an example simulation run was shown to participants to demonstrate the idea of what results of a simulation model look like. Participants worked in small groups looking at sections of the model printed on large paper (“Model Review” script [36, 41]) to suggest changes to the model. Additionally, participants were asked to suggest potential data sources for the model.

After working in small groups on the parts of the model, the group worked together on a stock and flow diagram representing the full model to integrate their suggested changes. Changes were made iteratively until all participants agreed the model accurately reflected community understanding of the problem. To conclude the workshop, participants suggested key areas where action was needed to improve water consumption and decrease SSB consumption.

#### Third workshop

After the second workshop, the revisions to the model were parametrised. The suggested local data on taste, acceptability, and accessibility from routine water authority surveys and publicly available public health data were used to further refine the parameters in the model.

The third iteration of the model was presented to stakeholders comprising six sections: SSB addiction and norms, water taste, water norms, public health, and access to public water. These sections were presented as feedback loops that interact with one another to ultimately influence water and SSB consumption. Additionally, simulation model runs showing the impact individually of role modelling (strengthening the water norm feedback look and weakening the SSB norm feedback loop), marketing (decreasing people’s overestimation of others’ consumption of SSB), and water taste (improving the taste of the water) were presented to the participants. The participants were then shown how combining interventions did not simply result in a change equal to the sum of the individual actions, but rather the interventions reinforced one another and created an even bigger change. The workshop concluded with the “Action Ideas” script [36] to brainstorm actions to increase local water consumption based on the insights generated from the modelling exercises, group conversations, and discussion about the implications of model runs. A full listing of the equations of the model can be found in Additional file 1.

#### Ethical approval for participation

This study design was approved by Deakin University Human Research Ethics Committee (HEAG-H 155_2014). Workshop participants received a plain language statement and consent form to sign prior to participation.

## Results

**Fig. 1:**
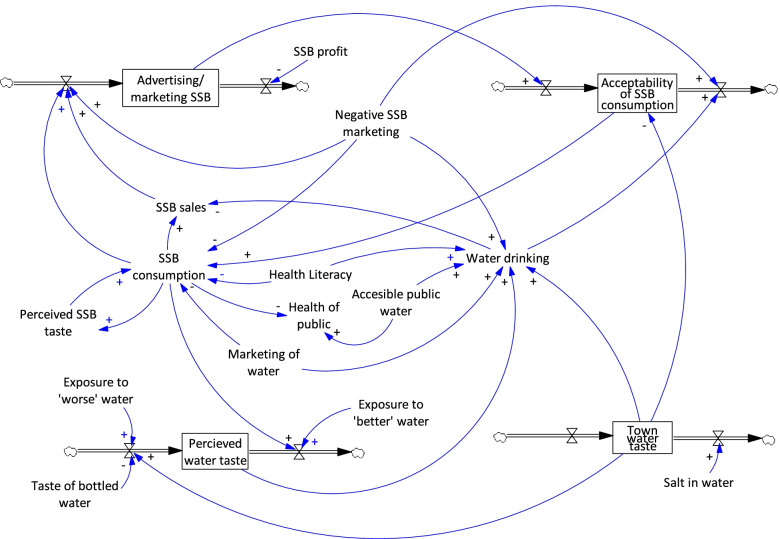
Initial qualitative stock and flow diagram

The first workshop developed the initial qualitative stock and flow diagram (Fig. 1 and Table 1) and four key components of the problem were identified as stocks: town water taste, perceived town water taste, advertising/marketing SSB, and acceptability of SSB consumption. The participants identified that it was important to separate out actual town water taste from perceived town water taste to recognise the delay between changes to the water taste and the public’s perception of the water. Additionally, the group recognised the challenge of the strong feedback loop between marketing of SSBs and the acceptability of SSB consumption, where marketing drives consumption, but then profits from consumption drive further advertising.

**Table 1:** Key elements of a stock and flow diagram

Element	Definition	Example
Stock	Represented as a phrase in a box. An element in a system, either tangible or intangible, that accumulates or depletes over time.	Perceived water taste
Flow	Represented as a valve connected to the stock with a cloud on the end. The process by which a stock accumulates or depletes over time.	Flows are often labelled, but not in the initial workshop. Changes in perceived water taste (Fig. 2)
Auxiliary variable	Represented as a phrase without a box around it. An intermediate element of the system used to clarify a connection between two or more flows or the equation underlying a flow.	Health literacy
Positive causal arrow	Represented as a blue arrow with a plus sign at the end. A causal relationship where the variable at the beginning of the arrow causes the variable at the arrowhead to change in the same direction.	An improvement in town water taste causes an improvement in perceived water taste AND deterioration in town water taste causes deterioration in perceived water taste.
Negative causal arrow	Represented as a blue arrow with a minus sign at the end. A causal relationship where the variable at the beginning of the arrow causes the variable at the arrowhead to change in the opposite direction.	An increase in health literacy causes a decrease in SSB consumption AND a decrease in health literacy causes an increase in SSB consumption.
Colour Coding	Colour is often used in models to draw attention to feedback loops and themes. In this project, each main feedback loop was colour coded for visibility.	The feedback loop representing addiction to SSB is coloured red in Fig. 3.

To parametrise the model, several changes were needed after the first workshop. Changes were guided by the session outputs (graphs over time and initial stock and flow diagram) and notes. Where there was uncertainty about how to represent the ideas discussed in the session as a stock and flow diagram, SD principles and the academic literature were used to fill in gaps, and these changes were noted to be checked with the participants in future workshops.

**Fig. 2:**
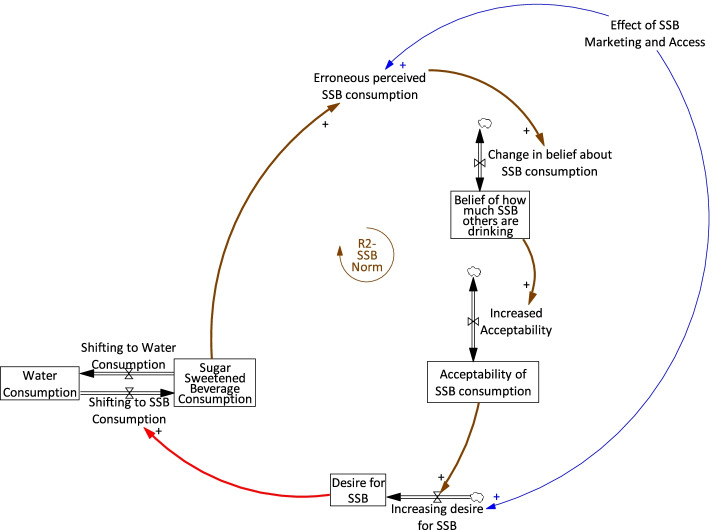
Qualitative representation of the sugar sweetened beverage norm section of the model

As one example, the modellers used the academic literature to operationalise the relationships between SSB consumption, advertising, and acceptability of SSBs (see Fig. 2 for relevant model section). Lally et al. [39] found that adolescents overestimate how much SSB their peers consume by 5.2 portions per week and how positively their peers view sugar sweetened beverages. This finding, supplemented by available public health data, was used to build a structure where actual SSB consumption was overestimated, leading to an overestimation in how acceptable it was to consume SSBs, and driving up SSB consumption over time. Marketing and access were conceptualised as a force that amplifies this effect. This synthesis of the causal relationships hypothesised by the participants and the evidence available in the literature was presented to the participants in the second workshop alongside the model diagram. Presenting literature in this way allowed participants to maintain ownership over the model, while also enhancing their own understanding of how the problem of SSB consumption may operate in the system from a feedback perspective. Further detail on data sources for the model can be found in Additional file 1.

**Fig. 3:**
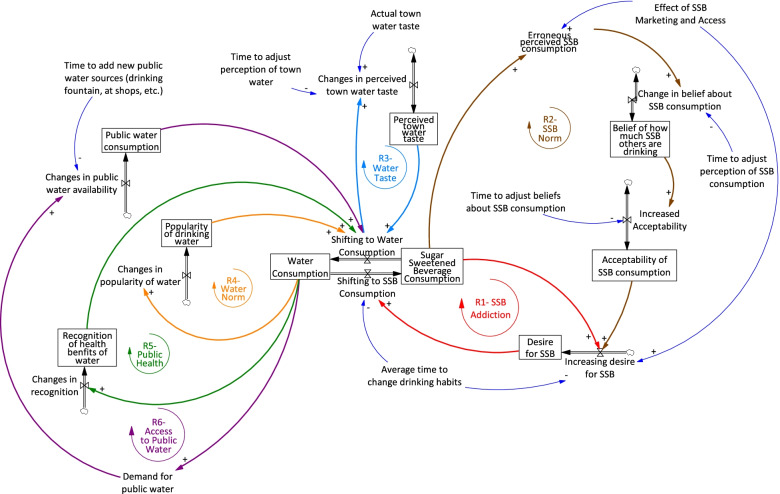
Qualitative stock and flow diagram representing the model resulting from the second workshop

The full model (Fig. 3) included six reinforcing feedback loops. Reinforcing loop 1 (R1), SSB addiction, represented the idea that as people drink more SSBs, their desire for SSBs grows, leading to further SSB consumption. SSB Norm (R2) represented the idea described above that people overestimate others’ SSB consumption, leading to norms encouraging further consumption. Water taste (R3) showed how as people tasted the water and did not like it, they drank less water, leading to further reduction in water consumption. Water norm (R4) indicated the degree to which drinking water was normalised in the community. Public health (R5) demonstrated the contribution that recognising the benefits of water consumption had to health outcomes. Finally, R6 (public water) reflected how availability of public water impacts water consumption, and therefore demand for more public water.

**Fig. 4:**
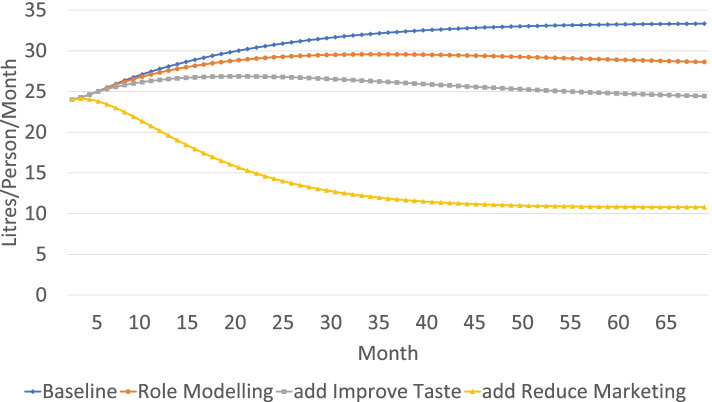
SSB consumption over time with varying interventions

While several data sources informed the model, it was beyond the scope of the workshops to fully validate the model and build confidence in the model’s ability to accurately indicate system behaviour that would result from the hypothesised relationships. Instead, the model served as a learning tool for participants to solidify their understanding of the interrelationships between the relevant parts of the system of interest and the resulting patterns of behaviour. Figure 4 presents a simulation model run presented to stakeholders. The graph shows that without intervention, SSB consumption remains high. Each other line shows the cumulative effect of adding one intervention on top of another. Ultimately, it shows that it is not the individual action of role modelling, improving the taste of the water, or reducing marketing that matters, but rather the combined effect of all three.

Even though the model outputs do not represent accurate outcomes of how SSB consumption would realistically change, the message of the importance of combining interventions had impact. A representative of the local water authority was present in the workshops, and he indicated that the use of simulation modelling demonstrated a serious commitment to public health by the stakeholders in the room and was one of the motivating factors that led to him exploring the possibility of changing the local taste of water. Ultimately, the stakeholders agreed to a pilot test of improving the taste of the water, combining both an improvement of water with a marketing and role modelling initiative. The participants reported that the modelling sessions were a key component of agreeing to the need for a combined intervention. The pilot at the local health service informed a larger piece of work to investigate and make the case for improving the water taste across Portland, paired with community engagement and health education. The process to improve water taste is ongoing, and the stakeholders from the workshop continue to engage in making this change possible.

## Discussion

### Major findings

This paper describes the development of a community-based SD model of the dynamics driving water and SSB consumption in the Portland community. This approach is one of the few published examples of active engagement of key stakeholders with significant authority to make change for healthier environments in modelling. This method helps to bridge the gap between community understandings of a specific health issue and the published scientific literature.

The model highlighted several key leverage points where local action could improve water and SSB consumption behaviours and quantified the impacts of each variable in the broader context of interrelationships in the community. In the study region, historically there has been polarising debate about the role of perceived poor taste of the local tap water in decreasing water consumption and increasing SSB consumption. The model highlights that simply changing the taste of tap water in isolation would be insufficient to improve community water consumption because other interacting feedback loops would reinforce SSB consumption, undermining positive change. Instead, combining improving the taste of water with complementary strategies such as role modelling water consumption and reducing marketing of SSBs would have greater impact.

### Comparison with international literature

These findings are supported by other SD models, which, for example, show that SSB taxes alone will not impact oral health without comprehensive sugar reduction policy [42], and that the right mix of key stakeholders is required to enhance the strength of the modelling process and subsequent outcomes [43]. This study reinforces the importance of having local leaders in the room during the modelling development process and offers an example of how the modelling process itself, as opposed to the outcomes of the model, can motivate stakeholders to work together for change at the community level.

### Strengths

Whilst SD modelling using GMB has been used in other public health problems [27, 28], there has been limited utilisation of these methods to address issues related to low water and high SSB consumption at the community level relative to the interest in public health.

A strength of this work is the engagement with local stakeholders and co-creation by engaging researchers, community stakeholders and grassroots community members. This study represents one of the first examples to apply systems methods and collaboratively define and explore problems, before developing intervention approaches most likely to yield significant population impact [15]. For example, the involvement of the local water authority improved model quality through provision of quantitative data relating to customer feedback on water taste. As a secondary consequence of this engagement and model outcomes, the process led the water authority to increase drinking fountains and trial multiple new ways to improve water taste (e.g. a new reverse osmosis plant for the local hospital).

### Weaknesses

Producing the community-based model was complex and time consuming for both the involved researchers and community members. Model development with community members required several iterations with the group during the workshops, along with extensive work by researchers between workshops. The process was dependent on continuous engagement from community members and the research team members’ high-level skills and expertise in SD modelling methods. This raises a question about whether the modelling that results from these methods truly builds community capacity in SD simulation modelling. This study process paid special attention to formulating and communicating the technical aspects of the water/SSB model to the level of expertise of community participants [44] with constant checking in about understandability of the process. In addition, the model development required strong facilitation stills to ensure the model was strengthened through integration of evidence whilst being consistent with community perceptions. Commitment to the issue at hand from both researchers and community members was a critical aspect of success. However, it is acknowledged that without this ongoing commitment and these specialised skills, it may be challenging to replicate these findings in another community.

Furthermore, the SD modelling process undertaken allowed several iterations of the model to ensure the model was rigorous and correctly represented the complexity of the issue in their community. Even with these iterations, the model was not developed to the point of providing realistic indications of behaviour over time. Instead, the focus of the modelling was on participants’ ideas and SD conventions to make it a learning exercise. Incorporating data that would build confidence in the model’s ability to reflect reality would have required further resources not available at the community level. This limitation highlights that SD modelling may be more feasible as a collaboration and learning tool in communities rather than a tool to predict likely outcomes of interventions.

The range of participants in the workshops has, to date, been limited with only a small number of organisations represented, although these participants had detailed knowledge to represent the broader community (e.g. the water authority had years of survey data, health specialists had years of experience working in community, etc.). Consequently, it is important to acknowledge that some relevant perceptions about connections between influences, outcomes, and health impacts may have been missed [15]. While attempting to collect and reflect dynamic interactions, SD models reflect and simulate mental models of participants at a particular point in time [45] and assume that the observed past dynamics and interactions will continue. Formal use of these models in intervention design would therefore benefit from constant refinement as both mental models and trial results provide more insight.

### Implications for practice

Many public health problems have remained resistant to change because the traditional single intervention approach is not enough to address their true complexity. In the current example, a campaign to encourage drinking more water would inevitably be hampered by perceived water taste and societal norms, and without identifying and addressing these influences, would most likely result in limited shifts in behaviour. Innovation in new intervention methods, which actively target multiple proximal and distal behavioural drivers simultaneously are too easily dismissed due to a lack of evidence of effectiveness. Yet, multi-component trials will not be funded without some evidence that they are worth funding. The current methods provide the ability for community members to explicate their mental models of the system within their local community, integrate the best of the current evidence base about individual cause and effect and simulate multiple possible intervention scenarios prior to implementation.

The SD modelling still needs to be undertaken in combination with standard health promotion principles and best available evidence for impacts to be effective, sustainable, and equitable. SD modelling will often help identify the leverage points but will not always indicate the best form of action (e.g., the model may indicate the need to simultaneously address water taste and water drinking norms, but there are many ways norms could be changed that may have varying levels of effectiveness).

## Conclusions

Key stakeholders were engaged in the process of building a SD simulation model to provide insight into water and SSB consumption in one Australian community. The process increased engagement and collaboration to address the problem among community stakeholders.

## Supplementary Information

**Additional file 1.** Model equations. A full listing of the equations of the system dynamics model built with stakeholders.

## Data Availability

All data generated and analysed during the current study are published in this article, including a full listing of model equations in Additional file 1.

## Abbreviations

GMB: Group model building
SD: System dynamics
SSB: Sugar sweetened beverage
WHO STOPS: Whole of Systems Trial of Prevention Strategies for Childhood Obesity
