# Supplementary material for: System dynamics modelling to engage community stakeholders in addressing water and sugar sweetened beverage consumption
Source: Int J Behav Nutr Phys Act. 2022 Sep 10;19:118. doi: 10.1186/s12966-022-01363-4 (PMC9463801; doi:10.1186/s12966-022-01363-4)
Supplement: Supplementary file 1 — Additional file 1. Model equations. A full listing of the equations of the system dynamics model built with stakeholders. [file 12966_2022_1363_MOESM1_ESM.docx]

Water Model Equation Listing

Below is a list of all the equations in the model built with stakeholders in Portland, VIC, Australia to understand the drivers of water consumption. The equations are from the documentation produced by Vensim, but these equations can be used with any software package capable of system dynamics simulation modelling.

INITIAL TIME = 0

Units: Month

FINAL TIME = 100

Units: Month

TIME STEP = 0.0625

Units: Month

| **Variable name** | **Variable type** | **Units** | **Equation** | **Notes** |
| --- | --- | --- | --- | --- |
| Acceptability of SSB consumption | Stock | L/Month | INTEG (Increased Acceptability, 20) | The initial value of this stock (20 L/month) was set to be equal to the initial actual Sugar Sweetened Beverage Consumption in order to see over time how the acceptability changes relative to actual consumption as a result of the various feedback processes. |
| Increased Acceptability | Flow | L/(Month*Month) | (("Descriptive norm- belief about how much others are drinking"-Acceptability of SSB consumption)/Time to adjust beliefs about SSB consumption)*(Maximum desire for SSB-Acceptability of SSB consumption)/Maximum desire for SSB |  |
| "Descriptive norm- belief about how much others are drinking" | Stock | L/Month | INTEG (Change in belief about SSB consumption, 20) | The initial value of this stock (20 L/month) was set to be equal to the initial actual Sugar Sweetened Beverage Consumption in order to see over time how the norm changes relative to actual consumption as a result of the various feedback processes. |
| Change in belief about SSB consumption | Flow | L/Month/Month | Gap between perception and reality of SSB consumption/Time to adjust perception of SSB consumption |  |
| Desire for SSB | Stock | L/Month | INTEG (Increased Desire for SSB, 20) | The initial value of this stock (20 L/month) was set to be equal to the initial actual Sugar Sweetened Beverage Consumption in order to see over time how desire changes relative to actual consumption as a result of the various feedback processes. |
| Increased desire for SSB | Flow | L/(Month*Month) | (Fraction of current consumption that could change each month*Sugar Sweetened Beverage Consumption/(Average time to change drinking habits*Percentage of time delay for desire change))*((Maximum desire for SSB-Desire for SSB)/Maximum desire for SSB)*Effect of acceptability on desire for SSB (Acceptability of SSB consumption/Sugar Sweetened Beverage Consumption)*Effect of SSB Marketing and Access |  |
| Perceived water taste | Stock | Taste | INTEG (Changes in perceived water taste, 45) | The initial perceived water taste (on a scale of 0 – 100) was set based on surveys conducted by the local water authority. Sensitivity analyses ensured that the model behaved similarly within a reasonable range of values. |
| Changes in perceived water taste | Flow | Taste/Month | Gap between actual and perception/Time to adjust perception of water+Normal change in taste per month*Effect of water consumption on perceived taste(Shifting to Water Consumption/Maximum increase in water consumption per month) |  |
| Public water consumption | Stock | L/Month | INTEG (More water being made available publicly, 0.00666667) | Public water consumption primarily refers to drinking fountains in Portland, but may refer to free water sources available elsewhere. The initial estimate was set low (one-fifth of a litre per month) based on feedback from the group about the relative availability of drinking fountains and people’s inclination to drink water from these sources. Sensitivity analyses ensured that the model behaved similarly within a reasonable range of values. |
| More water being made available publicly | Flow | L/Month/Month | MAX(0,Gap between public water demand and public water supply/Average time to add more water publicly |  |
| Sugar Sweetened Beverage Consumption | Stock | L/Month | INTEG (Shifting to SSB Consumption-Shifting to Water Consumption, 20) | The initial value of this stock was set to a realistic value based on data from the Australian Bureau of Statistics on water consumption and sugar sweetened beverage consumption across groups [1], and then sensitivity analyses were carried out to test the ranges at which model behaviour shifted based on different starting values of consumption. |
| Shifting to SSB Consumption | Flow | L/Month/Month | Gap between desired and actual SSB consumption/(Average time to change drinking habits*Percentage of time delay for behavior change |  |
| Shifting to Water Consumption | Flow | L/Month/Month | More water being made available publicly*Effect of perceived water taste on water consumption (Perceived water taste/Maximum water taste) |  |
| Water Consumption | Stock | L/Month | INTEG (Shifting to Water Consumption-Shifting to SSB Consumption, 55) | The initial value of this stock was set to a realistic value based on data from the Australian Bureau of Statistics on water consumption and sugar sweetened beverage consumption across groups [1], and then sensitivity analyses were carried out to test the ranges at which model behaviour shifted based on different starting values of consumption. |
| Actual water taste | Auxiliary | Taste | 45 | Actual water taste was set to equal perceived water taste at the beginning of the simulation run to reflect that Portland’s water taste had not changed in a long time. |
| Average time to add more water publicly | Auxiliary | Month | 6 | The average time to add more water publicly was tied to how long on average it took to build a new water fountain. This estimate was based on available grey literature [2, 3] and confirmation from the local water authority. |
| Average time to change drinking habits | Auxiliary | Months | 1.33 | This estimate of how long it takes to change health behaviour habits was informed by various sources from the literature [4-7]. Given the uncertainty in habit formation, sensitivity analyses were carried out to ensure the model behaved the same within a range of behaviours. |
| Demand for public water | Auxiliary | L/Month | Water Consumption*Desired proportion of water drank in public |  |
| Desired proportion of water drank in public | Auxiliary | Dmnl | 0.07 | This number represented how much people water people wanted to drink from drinking fountains and other sources. This number was very difficult to estimate, so a realistic number was selected, followed by sensitivity analyses to test impact on model behaviour. |
| Effect of acceptability on desire for SSB | Table function | Dmnl | ([(0,0)-(2,3)],(0,0),(0.0978593,0.197368),(0.207951,0.407895),(0.409786,0.592105),(0.721713,0.842105),(1,1),(1.26605,1.42105),(1.37615,1.90789),(1.44343,2.32895),(1.6208,2.68421),(1.75535,2.82895),(2,3)) | This table function and the ones below represent nonlinear functions describing the relationship between two variables. The values in the square brackets represent the range of values that the table function is expected to take as an input. The remaining values represent points along the curve to define the output given in response to the input.  In the flow Increased desire for SSB, this function takes the Acceptability of SSB consumption normalised by Sugar sweetened beverage consumption to represent acceptable sugar sweetened beverage consumption as a percentage of actual sugar sweetened beverage consumption.  The shape of this function was informed by feedback from the group about how greater acceptability of SSB will drive greater consumption. |
| Effect of perceived water taste on water consumption | Table function | Dmnl | [(0,0)-(1,2)],(0,0),(0.0764526,0.0263158),(0.168196,0.0701754),(0.314985, 0.245614),(0.415902,0.421053),(0.474006,0.692982),(0.5,1),(0.562691,1.21053),(0.620795,1.4386),(0.697248,1.64912),(0.782875,1.86842),(0.813456,1.89474),(0.877676,1.9386),(1,2)) | In the flow Changes in perceived water taste, this function takes the Shifting to Water Consumption normalised by Maximum increase in water consumption per month to represent how much water consumption may increase relative to a change in perceived water taste relative to the maximum a person’s water taste may change in a month.  The shape of this function was informed by discussion with the group about how much a person’s consumption may realistically change in a given month relative to a change in water taste. This was supplemented by data from the water authority reflecting household water usage and ratings of the taste of water. |
| Effect of SSB Marketing and Access | Auxiliary | Dmnl | 2 | The total overestimation of SSB consumption was estimated from the literature [8, 9]. Based on conversations with the group, this number was then split between two variables, an overestimation and an effect of marketing and access, to reflect the group’s hypothesis that some of the overestimation comes from the relative availability and marketing of SSB. |
| Effect of water consumption on perceived taste | Table function | Dmnl | ( [(0,0)-(1,10)],(0,0),(0.1,1.1),(0.159021,2.36842),(0.229358,3.94737),(0.348624,6.92982),(0.394495,8.20175),(0.434251,9.3421),(0.495413,9.82456),(0.568807,9.47368),(0.654434,8.37719),(0.706422,6.22807),(0.727829,4.29825),(0.752294,3.15789),(0.785933,2.2807),(0.801223,1.22807),(1,1)) | In the flow Changes in perceived water taste, this function takes the Shifting to Water Consumption normalised by Maximum increase in water consumption per month to represent how much water consumption may increase relative to a change in perceived water taste relative to the maximum a person’s water taste may change in a month.  The shape of this function was informed by discussion with the group about how much a person’s consumption may realistically change in a given month relative to a change in water taste. This was supplemented by data from the water authority reflecting household water usage and ratings of the taste of water. |
| Erroneous perceived SSB consumption | Auxiliary | L/Month | Sugar Sweetened Beverage Consumption*Overestimation of SSB consumption |  |
| Fraction of current consumption that could change each month | Auxiliary | Dmnl | 0.001 | This number was derived from sensitivity analyses to reflect that water consumption in households is relatively stable over time based on data from the water authority, and even in the face of changing conditions (like water taste), consumption will still change gradually. |
| Gap between actual and perception | Auxiliary | Taste | Actual water taste-Perceived water taste |  |
| Gap between desired and actual SSB consumption | Auxiliary | L/Month | Desire for SSB-Sugar Sweetened Beverage Consumption |  |
| Gap between perception and reality of SSB consumption | Auxiliary | L/Month | Erroneous perceived SSB consumption-"Descriptive norm- belief about how much others are drinking" |  |
| Gap between public water demand and public water supply | Auxiliary | L/Month | Demand for public water-Public water consumption |  |
| Maximum desire for SSB | Auxiliary | L/Month | 75 | This value put a limit on how much SSB someone could possibly drink, informed by normal liquid consumption. |
| Maximum increase in water consumption per month | Auxiliary | L/Month/Month | 15 | This number was derived from sensitivity analyses to reflect that there are limits to how much more water someone will drink in a given month, even when interventions are in place. |
| Maximum water taste | Auxiliary | Taste | 100 | This value was used to normalise the value of the taste of water so it could be a scale of 0-100. |
| Normal change in taste per month | Auxiliary | Taste/Month | 1 | This value was used as an input in the equation to determine how much perceived water taste could change each month, informed by actual water taste, actual water consumption, and existing perceptions of the water taste. |
| Overestimation of SSB consumption | Auxiliary | Dmnl | 1.025*Effect of SSB Marketing and Access | The total overestimation of SSB consumption was estimated from the literature [8, 9]. Based on conversations with the group, this number was then split between two variables, an overestimation and an effect of marketing and access, to reflect the group’s hypothesis that some of the overestimation comes from the relative availability and marketing of SSB. |
| Percentage of time delay for behaviour change | Auxiliary | Dmnl | 0.5 | These two parameters just made the point that it takes time first to get a message out and change people’s desires, then for behaviour to actually change. This made the point that there may be no change in behaviour at all at first as people’s desires change, followed by a gradual behaviour change. These numbers were informed by sensitivity analyses to ensure reasonable model behaviour. |
| Percentage of time delay for desire change | Auxiliary | Dmnl | 0.5 |  |
| Time to adjust beliefs about SSB consumption | Auxiliary | Month | 6 | While there have been numerous studies on the effects of beliefs, perceptions, and norms on health habits, it was difficult to find studies that specifically investigated time for these to change. Therefore, some initial literature [10, 11] was combined with discussion amongst the group and sensitivity analyses to create a reasonable estimate. Emphasis was placed on the uncertainty of these numbers, while focusing discussion on the fact that time was needed to allow beliefs, perceptions, and norms to change. |
| Time to adjust perception of SSB consumption | Auxiliary | Month | 6 |  |
| Time to adjust perception of water | Auxiliary | Month | 6 |  |

1. Australian Bureau of Statistics. National Health Survey: First results 2022. Available from: <https://www.abs.gov.au/statistics/health/health-conditions-and-risks/national-health-survey-first-results/latest-release>.

2. Bottled Water Alliance. The Manly Council water fountain project: How councils can reinvest the water fountain. 2010.

3. Victorian Health Promotion Foundation. Provision of drinking water fountains in public areas: A local government action guide. 2015.

4. Gardner B, Lally P, Wardle J. Making health habitual: the psychology of ‘habit-formation’ and general practice. British Journal of General Practice. 2012;62(605):664-6.

5. Fjeldsoe B, Neuhaus M, Winkler E, Eakin E. Systematic review of maintenance of behavior change following physical activity and dietary interventions. Health Psychology. 2011;30(1):99.

6. Gardner B, de Bruijn G-J, Lally P. A Systematic Review and Meta-analysis of Applications of the Self-Report Habit Index to Nutrition and Physical Activity Behaviours. Annals of Behavioral Medicine. 2011;42(2):174-87.

7. Kwasnicka D, Dombrowski SU, White M, Sniehotta F. Theoretical explanations for maintenance of behaviour change: a systematic review of behaviour theories. Health Psychology Review. 2016;10(3):277-96.

8. Lally P, Bartle N, Wardle J. Social norms and diet in adolescents. Appetite. 2011;57(3):623-7.

9. Lally P, van Jaarsveld CHM, Potts HWW, Wardle J. How are habits formed: Modelling habit formation in the real world. European Journal of Social Psychology. 2010;40(6):998-1009.

10. Robinson E, Harris E, Thomas J, Aveyard P, Higgs S. Reducing high calorie snack food in young adults: a role for social norms and health based messages. International Journal of Behavioral Nutrition and Physical Activity. 2013;10(1):73.

11. Stok FM, de Vet E, de Ridder DTD, de Wit JBF. The potential of peer social norms to shape food intake in adolescents and young adults: a systematic review of effects and moderators. Health Psychology Review. 2016;10(3):326-40.
